# White Matter Abnormalities in Children with HIV Infection and Exposure

**DOI:** 10.3389/fnana.2017.00088

**Published:** 2017-09-29

**Authors:** Marcin Jankiewicz, Martha J. Holmes, Paul A. Taylor, Mark F. Cotton, Barbara Laughton, André J. W. van der Kouwe, Ernesta M. Meintjes

**Affiliations:** ^1^Division of Biomedical Engineering, Department of Human Biology, University of Cape Town, Cape Town, South Africa; ^2^Scientific and Statistical Computing Core, National Institutes of Health, Bethesda, MD, United States; ^3^Stellenbosch University, Stellenbosch, South Africa; ^4^Athinoula A. Martinos Center for Biomedical Imaging, Massachusetts General Hospital, Charlestown, MA, United States

**Keywords:** HIV, DTI, HIV exposure, HIV infection, children

## Abstract

**Background:** Due to changes in guidelines and access to treatment, more children start combination antiretroviral therapy (ART) in infancy. With few studies examining the long-term effects of perinatal HIV infection and early ART on neurodevelopment, much is still unknown about brain maturation in the presence of HIV and ART. Follow-up studies of HIV infected (HIV+) children are important for monitoring brain development in the presence of HIV infection and ART.

**Methods:** We use diffusion tensor imaging (DTI) to examine white matter (WM) in 65 HIV+ and 46 control (HIV exposed uninfected (HEU) and HIV unexposed uninfected (HU)) 7-year-old children. This is a follow up of a cohort studied at 5 years, where we previously reported lower fractional anisotropy (FA) in corticospinal tract (CST) and mean diffusivity (MD) increases in inferior/superior longitudinal fasciculi (ILF/SLF), inferior fronto-occipital fasciculus (IFOF) and uncinate fasciculus (UF) in HIV+ children compared to uninfected controls. In addition, we also found a difference in FA related to age at which ART was initiated.

**Results:** At 7 years, we found two regions in the left IFOF and left ILF with lower FA in HIV+ children compared to controls. Higher MD was observed in a similar region in the IFOF, albeit bilaterally, as well as multiple clusters bilaterally in the superior corona radiata (SCR), the anterior thalamic radiation (ATR) and the right forceps minor. Unlike at 5 years, we found no impact on WM of ART initiation. In HEU children, we found a cluster in the right posterior corona radiata with higher FA compared to HU children, while bilateral regions in the CST demonstrated reduced MD.

**Conclusions:** At age 7, despite early ART and viral load (VL) suppression, we continue to observe differences in WM integrity. WM damage observed at age 5 years persists, and new damage is evident. The continued observation of regions with lower FA and higher MD in HIV+ children point to disruptions in ongoing white matter development regardless of early ART. Lastly, in HEU children we find higher FA and lower MD in clusters in the CST tract suggesting that perinatal HIV/ART exposure has a long-term impact on WM development.

## 1. Introduction

The treatment of childhood HIV infection has changed dramatically over the past 10 years. Changes in guidelines and access to treatment have increased the number of children beginning combination antiretroviral (ARV) therapy (ART) in infancy. The introduction of ART in infancy and early childhood may influence the effect of the virus, and may also have a direct effect on the child's maturing central nervous system. Since few studies have examined the long-term effects of perinatal HIV infection and early ART on neurodevelopment, little is known about brain maturation in the presence of HIV and ART. Neuroimaging studies have identified HIV associated differences in children, including regional and global white matter (WM) atrophy (van Arnhem et al., 2013; Sarma et al., 2014; Cohen et al., 2016; Yadav et al., 2017). Although WM continues developing well into adulthood (Reiss et al., 1996), the greatest increases in organization are typically within the first 10 years of growth (e.g., Bashat et al., 2005; Lebel et al., 2008; Lebel and Beaulieu, 2011), highlighting the importance of identifying damage or delayed development before adolescence.

Diffusion tensor imaging (DTI) is a non-invasive MRI technique that provides quantitative measures of WM microstructure. In childhood, fractional anisotropy (FA) typically increases with age and has been associated with increased myelination, greater axonal count and axonal density (Giedd et al., 1999; Filippi et al., 2002; Barnea-Goraly et al., 2005; Brouwer et al., 2012; Lentz et al., 2014). Additional DTI measures include mean diffusivity (MD), axial diffusivity (AD), and radial diffusivity (RD). MD is associated with structural organization, with lower values indicating well-organized structure and denser axonal packing (Feldman et al., 2010). In children RD values tend to decrease with age, which is interpreted as representing increased myelination and/or more densely packed axons; conversely, AD values tend to increase with age, which has been interpreted as improved fiber coherence as axons straighten and merge within the bundle (Lebel and Beaulieu, 2011).

DTI studies in HIV infected (HIV+) children and adolescents on ART have found lower whole brain (Uban et al., 2015) and regional FA, including the corpus callosum (CC), fornix, corona radiata, frontal and parietal WM, pre-/postcentral gyrus, and superior longitudinal fasciculus (Hoare et al., 2015; Li et al., 2015). Reductions in FA were attributed to RD increases, suggesting effects of HIV on myelination. However, most children in these studies initiated ART after 2 years of age, according to concurrent guidelines or delayed diagnosis. Although early ART is recommended to limit the effects of HIV infection in children, it is not yet known to what extent early ART can prevent or reverse HIV-associated WM damage.

Here we present DTI results in HIV+ children at age 7 years (7.01–7.84 years) from the CHER (Children with HIV Early Antiretroviral Therapy) clinical trial (Violari et al., 2008; Cotton et al., 2013) in follow-up at the Family Infectious Diseases Clinical Research Unit, Tygerberg Children's Hospital, Cape Town. These children initiated ART before 18 months of age and have been followed since enrollment under 12 weeks of age. We have previously reported lower FA in corticospinal tract and MD increases in inferior/superior longitudinal fasciculi (ILF/SLF), inferior fronto-occipital fasciculus (IFOF), and uncinate fasciculus (UF) in these children compared to uninfected controls at age 5 years (Ackermann et al., 2016), indicating that early ART may not fully protect WM development. Follow-up studies of infected children are particularly important for monitoring brain maturation in the presence of both HIV infection and ART, and the results can be used to identify critical time points for intervention.

Importantly, compared to Ackermann's study (Ackermann et al., 2016), the present study has an increased sample size and a group of HIV exposed, uninfected (HEU) children. While HEU children tend to be healthier than HIV+ children, studies find increased rates of infections and developmental delays compared to their HIV unexposed uninfected (HU) peers. Due to the increased success of preventing mother to child transmission (PMTCT) programs, there is a growing population of HEU children in regions with high HIV prevalence, such as South Africa. It is unclear if the reasons behind the increased risks are related to maternal HIV infection, perinatal ART exposure, environmental factors or a combination thereof. Two recent studies used DTI to study HIV exposure, with one finding no exposure differences (Jahanshad et al., 2015) while the other identified a region in which HEU infants had higher FA (Tran et al., 2016) then infants.

Here we examine group differences in WM integrity between HIV+ and uninfected children at age 7 years, and between HEU and HU children. In addition, within the HIV+ children we look at possible treatment differences between those who initiated ART before and after 12 weeks of age. Based on previous results from cohort at an earlier age and on the other studies in the literature, we hypothesized that HIV+ children would demonstrate several WM regions of reduced FA and increased MD compared to uninfected controls, while comparisons of HEU to HU children would not reveal any differences. Further, we also hypothesized that children who initiated ART before 12 weeks of age would show fewer WM alterations compared to controls than those who started ART later.

## 2. Materials and methods

### 2.1. Participants

Participants were 72 Xhosa and Cape Colored 7-year old HIV+ children from the CHER trial (Violari et al., 2008; Cotton et al., 2013) and 56 uninfected age-matched controls from the same community recruited as part of a parallel vaccine study (Madhi et al., 2010). As part of the CHER trial, infants with CD4 percentage (CD4%) of at least 25% were randomized to one of the following three treatment arms: ART-Def (ART deferred until CD4% < 25% in first year or CD4% < 20% thereafter, or if clinical disease progression criteria presented); ART-40W (ART initiated before 12 weeks of age and interrupted after 40 weeks); and ART-96W (ART initiated before 12 weeks of age and interrupted after 96 weeks). ART was restarted for any child in the in ART-40W and ART-96W groups if CD4% declined or clinical evidence of disease progression was observed. Since several children in the ART-Def arm met criteria for almost immediate initiation of ART, the children were grouped here based on age at treatment initiation, specifically those who received ART at or before 12 weeks (before-12wk) and those who received treatment after 12 weeks (after-12wk).

### 2.2. Image acquisition

All children were scanned without sedation on a 3 Tesla Siemens Allegra MRI (Erlangen, Germany) at the Cape Universities Brain Imaging Centre (CUBIC) in South Africa with a single channel head coil according to protocols that had been approved by the Human Research Ethics Committees of the participating institutions. Parents/guardians provided written informed consent and children oral assent. Children were first familiarized with the scanning procedures on a mock scanner.

For each child we acquired a structural T1-weighted (T1w) volume using a volumetric navigated (Tisdall et al., 2012) multiecho magnetization prepared rapid gradient echo (MEMPRAGE) sequence (van der Kouwe et al., 2008) (voxel size = 1.3 × 1.0 × 1.0 mm^3^, FOV = 224 × 224 × 144 mm^3^, TR = 2,530 ms, TI = 1,160 ms, TEs = 1.53/3.19/4.86/6.53 ms, flip angle = 7°) and a pair of diffusion weighted data sets with opposite phase encodings (here, anterior-posterior and posterior-anterior, AP-PA) for EPI distortion correction during processing (Irfanoglu et al., 2012) using a volumetric navigated (Alhamud et al., 2012) twice-refocused spin echo sequence (voxel size = 2.0 × 2.0 × 2.0 mm^3^, matrix size = 112 × 112 × 72, FOV = 224 × 224 × 144 mm^3^, TR/TE = 10,000/86 ms, 30 non-collinear gradient directions with DW factor *b* = 1, 000 s mm^−2^, and four non-DW *b* = 0 s mm^−2^ (*b*_0_) acquisitions).

### 2.3. Image preprocessing

Structural and DWI images were converted from the DICOM format to NiFTi format using the dcm2nii tool (http://www.cabiatl.com/mricro). The DWI data were first inspected visually for the presence of volumes with large motion artifacts or dropout slices, which were removed prior to processing. Motion-corrupted volumes were removed from both AP and PA acquisitions. Only subjects with more than 15 diffusion directions remaining were retained for subsequent analyses. DWI data were then corrected for motion, eddy current and EPI distortions using the DIFF_PREP and DR-BUDDI tools within TORTOISE v.2.5.2 (Pierpaoli et al., 2010; Irfanoglu et al., 2015). For the purposes of having a *b*_0_-like contrast for default TORTOISE processing, the inversion of the relative contrast of tissues (IRCT) method was applied to each subject's T1w volume to generate a T2w-like contrast volume (see Appendix A of Taylor et al., 2016); the T2w-like volumes were only used for registration within TORTOISE. After preprocessing, the diffusion tensors (DTs) and associated parameters (FA, MD, etc.) were computed using 3dDWItoDT in AFNI (Cox, 1996).

A standard space and WM mask for this study were created as follows. Each subject's T1w structural image was non-linearly warped to the 2 mm isotropic Haskins pediatric template using AFNI's 3dQwarp. The warping transformation was then applied to each of the DTI parameter volumes of interest. To generate a group-level WM mask based on the subject data, the coregistered FA maps of all subjects were thresholded at FA > 0.2 and then combined together as an intersection across the subjects in order to restrict analysis to WM (Mori and van Zijl, 2002). Voxelwise comparisons of DTI measures were only performed within this WM mask.

### 2.4. Voxelwise image analyses

Voxelwise group comparisons were performed in FSL based on a general linear model with randomize (Winkler et al., 2014) that included gender and ethnicity as confounds. Clusters where DTI parameters differed between groups were identified from uncorrected *p*-value maps for 3 sets of comparisons: HIV+ vs. Controls; Before-12wk vs. After-12wk; and HEU vs. HU. To control for Type I error, Monte-Carlo simulations were performed using AFNI-3dClustsim (Forman et al., 1995). We set our cluster size threshold at *p*_*th*_ = 0.005 and α = 0.05 (nearest neighbors NN = 3, two-sided voxelwise thresholding), which yielded a minimum cluster size of 112 mm^3^ for Controls vs. HIV+, and 120 mm^3^ for the HEU vs. HU and before- and after-12 wk group comparisons.

## 3. Results

Data from 17 children (7 HIV+/10 controls) were excluded due to incomplete scans or the presence of motion artifacts in more than half the DTI volumes. After exclusions, we present data from 65 HIV+ children (51 before-12wk; 14 after-12wk) and 46 uninfected controls (19 HEU; 27 HU) (Table 1). Notably, in all except 9 children, VL was suppressed by age 2 years. At the time of scan, VL was suppressed in all but 3 HIV+ children (95%).

**Table 1:** Sample characteristics of Controls (HEU, HIV exposed uninfected; HU, HIV unexposed uninfected) and the HIV+ group (before-12wk, after-12wk).

**Demographics**	**Controls**		
	**All**	**HEU**	**HU**
Number of subjects (N)	46	19	27
Sex (M:F)	25:21	11:8	14:13
Ethnicity (Xhosa:Cape Colored)	37:9	18:1	19:8
Mean age at scan (yrs)	7.3 ± 0.1	7.2 ± 0.1	7.3 ± 0.2
**Demographics**	**HIV**		
	**All**	**Before-12wk**	**After-12wk**
Number of subjects (N)	65	51	14
Sex (M:F)	30:35	23:28	7:7
Ethnicity (Xhosa:Cape Colored)	56:9	44:7	12:2
Mean age at scan (yrs)	7.2 ± 0.1	7.2 ± 0.1	7.2 ± 0.2
**Clinical measures at baseline (6–8 weeks)**			
CD4 count (cells/mm^3^)		1, 744 ± 993	2, 056 ± 688
CD4% (%)		32 ± 11	36 ± 9
CD4/CD8		1.27 ± 0.76^a^	1.26 ± 0.67
CD8 count (cells/mm^3^)		1, 765 ± 1, 189^a^	1, 902 ± 952
CD8% (%)		31 ± 11^a^	32 ± 11
VL (>750,000) (N)		31	7
Low VL (400−750, 000) (N)		20	7
Suppressed VL (<400) (N)		0	0
**Clinical measures at scan (7 years)**			
CD4 count (cells/mm^3^)		1, 102 ± 447	1, 208 ± 527
CD4% (%)		37 ± 6	37 ± 7
High VL (>750,000) (N)		0	0
Low VL (400−750, 000) (N)		3	0
Suppressed VL (<400) (N)		48	14
**Other measures**			
Age at ART initiation (weeks)		8.55 ± 1.65	34.75 ± 18.07
Age at ART interruption (weeks)		71.31 ± 27.73^b^	–
Duration of ART interruption (weeks)		65.26 ± 88.89^b^	–
Cumulative duration on ART (weeks)		324.06 ± 69.93	341.85 ± 19.28
Age at first VL suppression (weeks)		33.43 (19.07)^c^	47.29 (34.71)^c^
CDC classification			
A (N)		6	0
B (N)		9	1
C (N)		27	8
S (Severe) (N)		9	5
HIV encephalopathy diagnoses		7	1

*Values are mean ± standard-deviation; VL, plasma viral load (RNA copies/ml)*.

a *Data were not available for 6 children*.

b *Age and duration of ART interruption mean and standard deviation based only on children in whom treatment was interrupted (N = 36)*.

c *Median and interquartile range*.

### 3.1. Controls vs. HIV+

**Figure 1:**
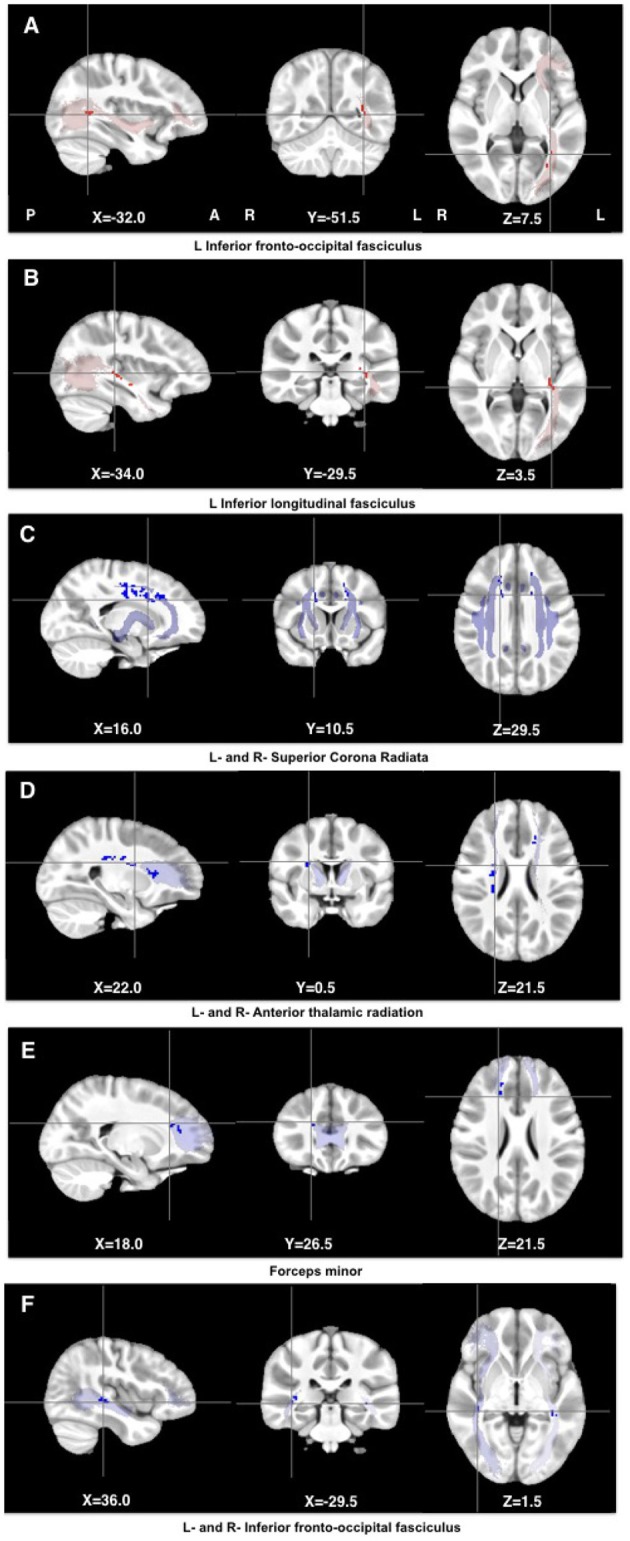
Clusters showing lower FA (red) and higher MD (blue) in HIV infected children compared to controls. The clusters were overlayed on masks of the corresponding tracts (JHU White-Matter Tractography Atlas).

Two regions in the left inferior fronto-occipital fasciculus (IFOF) and left inferior longitudinal fasciculus (ILF), respectively, showed lower FA in HIV+ children compared to controls. Higher MD was found in a similar region in the inferior fronto-occipital fasciculus (IFOF), albeit bilaterally, as well as multiple clusters bilaterally in the superior corona radiata and the anterior thalamic radiation (ATR), and right forceps minor (Table 2 and Figure 1). These FA decreases and MD increases were largely attributable to higher RD in HIV+ children.

**Table 2:** Peak (top) and center of gravity (COG) (bottom) MNI coordinates of clusters showing significant differences in FA or MD between Controls and HIV+ children.

**Cluster location** **peak** **COG**	**Size** **(mm^3^)**	**FA**			**AD**			**RD**		
		**Controls**	**HIV+**	***p***	**Controls**	**HIV+**	***p***	**Controls**	**HIV+**	***p***
L Inferior fronto-occipital fasciculus (−32.0, −51.5, 7.5) (−29.4, −52.6, 13.9)	344	0.62 (0.05)	0.55 (0.06)	<0.001	1.58 (0.10)	1.53 (0.09)	0.01	0.52 (0.06)	0.59 (0.07)	<0.001
L Inferior longitudinal fasciculus (−34.0, −29.5, 3.5) (−31.7, 24.6, 3.3)	304	0.58 (0.04)	0.53 (0.05)	<0.001	1.51 (0.07)	1.47 (0.07)	0.001	0.54 (0.04)	0.59 (0.04)	<0.001
**Cluster location** **peak** **COG**	**Size** **(mm^3^)**	**MD**			**AD**			**RD**		
		**Controls**	**HIV+**	***p***	**Controls**	**HIV+**	***p***	**Controls**	**HIV+**	***p***
R Superior corona radiata (16.0, 10.5, 29.5) (16.2, −2.5, 35.7)	576	0.79 (0.03)	0.82 (0.03)	<0.001	1.30 (0.06)	1.33 (0.06)	0.05	0.53 (0.04)	0.57 (0.04)	<0.001
L Superior corona radiata (−18.0, 4.5, 33.5) (−16.4, 4.2, 38.2)	368	0.80 (0.03)	0.83 (0.03)	<0.001	1.32 (0.05)	1.35 (0.06)	0.008	0.56 (0.05)	0.57 (0.04)	0.25
R Superior corona radiata (18.0, 24.5, 29.5) (16.1, 23.6, 31.9)	216	0.78 (0.03)	0.82 (0.04)	<0.001	1.31 (0.10)	1.34 (0.08)	0.12	0.52 (0.04)	0.56 (0.06)	<0.001
L Superior corona radiata (−18.0, −27.5, 41.5) (−19.8, −17.4, 39.3)	200	0.76 (0.03)	0.79 (0.03)	<0.001	1.28 (0.06)	1.31 (0.07)	0.01	0.50 (0.04)	0.52 (0.04)	0.005
L Superior corona radiata (−18.0, −28.5, 25.5) (−16.9, 27.4, 25.8)	112	0.80 (0.04)	0.83 (0.04)	<0.001	1.30 (0.08)	1.31 (0.07)	0.32	0.54 (0.04)	0.59 (0.05)	<0.001
R Anterior thalamic radiation (22.0, 0.5, 21.5) (22.2, −15.1, 26.4)	520	0.76 (0.02)	0.79 (0.03)	<0.001	1.19 (0.04)	1.23 (0.06)	<0.001	0.55 (0.03)	0.57 (0.03)	<0.001
L Anterior thalamic radiation (−28.0, −37.5, 9.5) (−28.5, −45.3, 12.1)	152	0.68 (0.07)	0.77 (0.08)	<0.001	1.24 (0.09)	1.34 (0.12)	<0.001	0.40 (0.08)	0.48 (0.08)	<0.001
L Anterior thalamic radiation (−18.0, 36.5, −2.5) (−17.2, 37.4, −4.0)	136	0.83 (0.04)	0.87 (0.04)	<0.001	1.38 (0.08)	1.40 (0.08)	0.20	0.55 (0.05)	0.60 (0.06)	<0.001
R Anterior thalamic radiation (22.0, 18.5, 11.5) (22.1, 18.4, 13.4)	128	0.76 (0.06)	0.80 (0.05)	<0.001	1.27 (0.06)	1.31 (0.07)	0.01	0.51 (0.05)	0.54 (0.06)	<0.001
L Anterior thalamic radiation (−22.0, −29.5, 27.5) (−23.9, −26.8, 25.4)	112	0.77 (0.03)	0.81 (0.04)	<0.001	1.28 (0.08)	1.29 (0.09)	0.32	0.52 (0.05)	0.56 (0.07)	<0.001
L Anterior thalamic radiation (−24.0, 10.5, 11.5) (−22.4, 14.6, 12.5)	112	0.74 (0.03)	0.77 (0.03)	<0.001	1.24 (0.06)	1.27 (0.07)	0.005	0.49 (0.04)	0.53 (0.06)	<0.001
R Forceps minor (18.0, 26.5, 21.5) (16.4, 33.0, 20.9)	224	0.81 (0.03)	0.84 (0.03)	<0.001	1.31 (0.07)	1.33 (0.08)	0.15	0.56 (0.05)	0.60 (0.05)	<0.001
R Forceps minor (16.0, 40.5, −0.5) (16.5, 44.2, 1.8)	200	0.85 (0.05)	0.89 (0.05)	<0.001	1.38 (0.07)	1.41 (0.08)	0.01	0.59 (0.05)	0.64 (0.06)	<0.001
R Inferior fronto-occipital fasciculus (36.0, −29.5, 1.5) (33.4, −31.2, 7.3)	200	0.87 (0.03)	0.91 (0.04)	<0.001	1.47 (0.06)	1.50 (0.07)	0.02	0.57 (0.04)	0.61 (0.04)	<0.001
L Inferior fronto-occipital fasciculus (−34.0, −35.5, 5.5) (−33.9, −33.8, 4.9)	128	0.87 (0.04)	0.91 (0.04)	<0.001	1.52 (0.06)	1.55 (0.08)	0.08	0.55 (0.05)	0.60 (0.06)	<0.001

*Group means (stdev) of FA and MD are shown, as well as AD and RD, for each cluster. Units of MD, AD, and RD are 10^−3^mm^2^s^−1^. Values are Mean (SD)*.

### 3.2. Before-12wk vs. after-12wk

No regions showed FA or MD differences based on when treatment was initiated. To confirm that the potential benefit of earlier treatment in the before-12wk children group was not compromised by interruption, we also compared the after-12wk group separately to children in the before-12wk group on continuous and interrupted treatment, respectively, and examined associations of FA with age of ART initiation adjusting for sex, ethnicity and duration of interruption. No regions showed significant effects for any of these analyses.

### 3.3. HEU vs. HU

**Figure 2:**
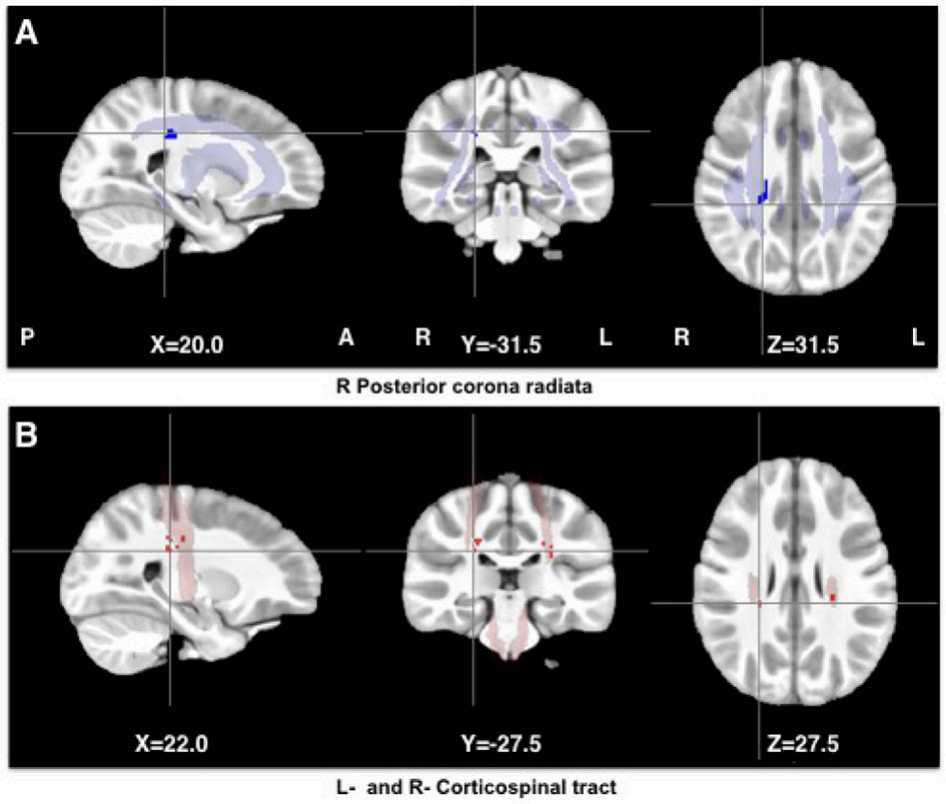
Clusters showing higher FA (blue) and lower MD (red) in HIV exposed uninfected (HEU) children compared to unexposed uninfected (HU) children. The clusters were overlayed on masks of the corresponding tracts (JHU White-Matter Tractography Atlas).

A cluster in the right posterior corona radiata had higher FA in HEU children than in HU children, while regions in the right- and left-corticospinal tract showed lower MD (Table 3 and Figure 2).

**Table 3:** Peak (top) and center of gravity (COG) (bottom) MNI coordinates of clusters showing significant differences in FA or MD between HU and HEU children.

**Cluster location** **peak** **COG**	**Size** **(mm^3^)**	**FA**			**AD**			**RD**		
		**HU**	**HEU**	***p***	**HU**	**HEU**	***p***	**HU**	**HEU**	***p***
R Posterior corona radiata (20.0, −31.5, 31.5) (19.1, −25.6, 33.0)	200	0.44 (0.04)	0.52 (0.06)	<0.001	1.31 (0.07)	1.37 (0.08)	0.02	0.63 (0.05)	0.56 (0.05)	<0.001
**Cluster location** **peak** **COG**	**Size** **(mm^3^)**	**MD**			**AD**			**RD**		
		**HU**	**HEU**	***p***	**HU**	**HEU**	***p***	**HU**	**HEU**	***p***
R Corticospinal tract (22.0, −27.5, 27.5) (18.1, −25.8, 31.9)	320	0.87 (0.05)	0.82 (0.03)	<0.001	1.37 (0.08)	0.12	1.34 (0.06)	0.63 (0.05)	0.56 (0.05)	<0.001
L Corticospinal tract (−26.0, −25.5, 33.5) (−25.6, −26.0, 28.6)	120	0.80 (0.03)	0.76 (0.02)	<0.001	1.22 (0.08)	1.14 (0.06)	<0.001	0.59 (0.04)	0.57 (0.03)	0.03

*Group means (stdev) of FA and MD are shown, as well as AD and RD, for each cluster. Units of MD, AD, and RD are 10^−3^mm^2^s^−1^. Values are Mean (SD)*.

## 4. Discussion

This study presents DTI findings at age 7 years in an expanded group of children who had been scanned at age 5 (Ackermann et al., 2016). Similar to earlier findings, our results point to alterations in WM microstructure in the presence of HIV infection despite early ART. Numerous regions were identified with lower FA or higher MD in HIV infected children compared to their uninfected peers. Although AD showed significant group differences in most regions, similar to the findings at 5 years, effects were largely attributable to RD increases in infected children, pointing to regional myelin damage, reduced myelination or myelin loss (Alexander et al., 2007). Contrary to the results at 5 years, we did not find differences based on timing of treatment initiation suggesting that by age 7 there is no protective effect in WM from starting treatment before or after 12 weeks. In addition, our analysis here revealed one cluster with higher FA and two with lower MD in HEU children compared to HU children demonstrating differences related to ART/HIV exposure in children.

### 4.1. Controls vs. HIV+

In contrast to other studies reporting FA reductions in the corpus callosum (CC) in children and youths on ART (Hoare et al., 2015; Li et al., 2015), we continue, as at 5 years (Ackermann et al., 2016), to find no evidence of microstructural CC damage at age 7 years in these children who all initiated ART by 18 months of age. The lack of HIV associated WM damage in the CC suggests that early treatment is neuroprotective to the CC. Children in the studies reporting CC damage initiated ART when clinically indicated.

However, despite early ART and VL suppression in 86% of the children by age 2 years, we continue to observe diffuse differences in WM integrity. Similar to our findings at 5 years (Ackermann et al., 2016), we again find HIV associated WM damage in the IFOF, ILF, and forceps minor, indicating that damage in these tracts may occur early during infection and persist during childhood. This concurs with spectroscopy findings from the same cohort studied here at age 5 years, where basal ganglia metabolite levels (choline, NAA) were associated with CD4/CD8 at enrollment (Mbugua et al., 2016).

A recent study of HIV+ adolescents on ART showed that WM integrity in these same three tracts were associated with measures of HIV disease severity (Uban et al., 2015). In particular peak VL was associated with reduced FA in the right IFOF, which partially mediated the effect of higher peak VL on poorer working memory performance (Uban et al., 2015). In addition, higher peak VL was related to higher streamline count (i.e., the number of fiber bundles) in the left ILF, which the authors interpret as a compensatory mechanism to deal with the impact of HIV and/or ART in this region. We found no evidence of compensation in this tract, but a loss of WM integrity. Disease severity (nadir CD4%) was also associated with AD and MD in the forceps minor (Uban et al., 2015), a fiber bundle that connects the lateral and medial surfaces of the frontal lobes and crosses the midline via the genu of the corpus callosum, and is responsible for interhemispheric sensory and auditory connectivity.

In addition to the above regions, we find MD increases at age 7 years in several clusters bilaterally along the superior corona radiata (SCR) and ATR that were not evident at 5 years. Two of the regions in the SCR are in similar locations, albeit contralateral, to the parietal corticospinal tract (CST) cluster where lower FA was found at age 5 years. Other regions, specifically the uncinate fasciculus (UF) and internal capsule and brain stem regions of the CST, showed WM damage at 5 years (Ackermann et al., 2016) but not at 7 years. These findings suggest that WM developmental delay in some regions may resolve, while other regions (viz. the SCR and ATR) may be sensitive to ongoing HIV infection and/or ART exposure.

While we found HIV-related RD increases in the SCR, Hoare et al. (2015) reported RD increases in the anterior corona radiate in children aged 6–16 years on ART. In infected children aged 13–17 years on ART, Li et al. (2015) found lower FA in the superior and posterior corona radiata, frontal and parietal WM, pre-/postcentral gyrus, and superior longitudinal fasciculus (SLF), all due to RD increases.

### 4.2. Before-12wk vs. after-12wk

Previously, at age 5 years, one cluster was found in which the before-12wk group demonstrated lower FA compared to the after-12wk group (Ackermann et al., 2016). The difference was attributed to the children in the before-12wk group whose treatment was interrupted, pointing to possible harmful effects of treatment interruption. Here we did not observe any differences based on timing of treatment initiation, indicating that HIV associated damage occurs either very early during infection (for example, in the IFOF, ILF and forceps minor) and as such affects children initiated before and after 12 weeks similarly, or later in development (for example, SCR and ATR) when all children are impacted equally.

In contrast to our findings, Li et al. (2015) found that longer ART duration and earlier age of treatment initiation was associated with lower frontal FA in infected youths. In their study, children initiated treatment when clinically indicated. As such, sicker children, in whom one might expect the most WM damage, would have initiated ART earlier and would have been on ART for longer. In our study, children were randomized to receive ART before or after 12 weeks, so that timing of ART initiation is not related to disease severity. Notably, we observe increasing damage in frontal WM (viz. ATR) in our children from 5 to 7 years, which overlaps with the time when children in (Li et al., 2015) start to initiate ART (age of ART initiation: 50–190 months). These findings suggest that frontal WM may be more vulnerable to ongoing HIV infection over this period of development, or that an early insult negatively impacts later development.

### 4.3. HEU vs. HU

We found one cluster in the right posterior corona radiata showing higher FA, accompanied by higher AD and lower RD, in HEU children compared to HU children. As the white matter sheet of the corona radiata is one of the first cells to form in embryos, it is possible that the observed increased FA in this region is related to *in utero* exposure to HIV and/or ART. Regional increases in FA are typically interpreted as representing higher WM connectivity due to more densely packed axons, greater axon diameter or myelination. However, increased FA has been observed in pathology with differing explanations, such as accelerated maturation in autistic children (Bashat et al., 2005) and excessive, thick myelin in children with attention-deficit/hyperactivity disorder (ADHD) (Li et al., 2010). In HEU infants, a recent study identified a region with higher mean FA in the middle cerebellar peduncles, which the authors interpret as potentially corresponding to microscopic deficits or reductions in axons (Tran et al., 2016).

In addition, we found clusters bilaterally in the corticospinal tract that demonstrated lower MD, with lower AD and RD. MD is associated with structural organization, with lower values indicating well organized structure. Denser axonal packing is thought to be related to lower MD values. In children, RD values decrease with age and is interpreted as representing increased myelination and/or more densely packed axons (Lebel and Beaulieu, 2011). The corona radiata is associated with the corticospinal tract, pointing to a possible relationship between the clusters showing exposure effects.

While a recent neuroimaging study of HEU children (Jahanshad et al., 2015) did not detect any group differences in DTI measures, the authors reported that higher FA and lower MD were each associated with higher IQ scores in both HEU and HU children. These results support the interpretation of increased connectivity with higher FA and lower MD, and suggest an absence of WM damage or delayed development in HEU children.

Further work exploring the relationship between DTI measures with other neuroimaging modalities as well as neuropsychological performance may help better understand these results.

### 4.4. Strengths and limitations

The strength of our study is that the cohort has been followed from a young age and is well characterized. The relatively narrow age ranges over which imaging has been performed also facilitate a description of longitudinal changes. Unfortunately the cohort was not assessed for prenatal or perinatal HIV infection due to tests available at the time of birth. We also did not determine the effects of nutrition or other infections, e.g., cytomegalovirus.

## 5. Conclusions

This study presents a follow up of a cohort studied at 5 years, revealing ongoing WM alterations at age 7 years in HIV infected children compared to controls despite early ART and VL suppression. WM damage observed at age 5 years in the IFOF, ILF, forceps minor and CST persists. In addition, new WM damage is evident in multiple clusters along the SCR and ATR. The continued observation of clusters with lower FA and higher MD in HIV infected children point to disruptions in ongoing white matter development regardless of early ART. The fact that treatment initiation before or after 12 weeks does not influence WM integrity at this age further suggests that WM damage occurs either very early in infection or later in development when children initiating ART before and after 12 weeks are impacted similarly. In addition, in HEU children we find higher FA and lower MD in clusters in the CST suggesting that perinatal HIV/ART exposure has a long-term impact on WM development.

## Ethics statement

This study was carried out in accordance with the recommendations of Human Research Ethics Committees of the participating institutions with written informed consent from all subjects. All subjects gave written informed consent in accordance with the Declaration of Helsinki. The protocol was approved by the Human Research Ethics Committees of the participating institutions.

## Author contributions

MJ and PT were involved in designing and performing data analyses. EM, AvK, and BL conceived, designed and obtained funding for the study. MJ, MH, EM, BL, and MC provided interpretation of data for the work. MJ and MJH drafted the work and all other authors provided critical revision of the manuscript.

### Conflict of interest statement

The authors declare that the research was conducted in the absence of any commercial or financial relationships that could be construed as a potential conflict of interest.
